# Head impulse compensatory saccades: Visual dependence is most evident in bilateral vestibular loss

**DOI:** 10.1371/journal.pone.0227406

**Published:** 2020-01-15

**Authors:** Jacob M. Pogson, Rachael L. Taylor, Leigh A. McGarvie, Andrew P. Bradshaw, Mario D’Souza, Sean Flanagan, Jonathan Kong, G. Michael Halmagyi, Miriam S. Welgampola

**Affiliations:** 1 Royal Prince Alfred Hospital, Institute of Clinical Neuroscience, Camperdown, New South Wales, Australia; 2 Faculty of Health and Medicine, Sydney Medical School, The University of Sydney, Camperdown, New South Wales, Australia; 3 Department of Psychology, Faculty of Science, The University of Sydney, Camperdown, New South Wales, Australia; 4 Department of Clinical Research, Royal Prince Alfred Hospital, Camperdown, New South Wales, Australia; 5 Otolaryngology, Head and Neck and Skull Base Surgery, St Vincent’s Hospital, Darlinghurst, New South Wales, Australia; 6 Faculty of Medicine, University of NSW, Kensington, New South Wales, Australia; 7 Department of Neurosurgery, Royal Prince Alfred Hospital, Camperdown, New South Wales, Australia; 8 Department of Otolaryngology, Head & Neck Surgery, Royal Prince Alfred Hospital, Camperdown, New South Wales, Australia; University of Rochester, UNITED STATES

## Abstract

The normal vestibulo-ocular reflex (VOR) generates almost perfectly compensatory smooth eye movements during a ‘head-impulse’ rotation. An imperfect VOR gain provokes additional compensatory saccades to re-acquire an earth-fixed target. In the present study, we investigated vestibular and visual contributions on saccade production. Eye position and velocity during horizontal and vertical canal-plane head-impulses were recorded in the light and dark from 16 controls, 22 subjects after complete surgical unilateral vestibular deafferentation (UVD), eight subjects with idiopathic bilateral vestibular loss (BVL), and one subject after complete bilateral vestibular deafferentation (BVD). When impulses were delivered in the horizontal-canal plane, in complete darkness compared with light, first saccade frequency mean(SEM) reduced from 96.6(1.3)–62.3(8.9) % in BVL but only 98.3(0.6)–92.0(2.3) % in UVD; saccade amplitudes reduced from 7.0(0.5)–3.6(0.4) ° in BVL but were unchanged 6.2(0.3)–5.5(0.6) ° in UVD. In the dark, saccade latencies were prolonged in lesioned ears, from 168(8.4)–240(24.5) ms in BVL and 177(5.2)–196(5.7) ms in UVD; saccades became less clustered. In BVD, saccades were not completely abolished in the dark, but their amplitudes decreased from 7.3–3.0 ° and latencies became more variable. For unlesioned ears (controls and unlesioned ears of UVD), saccade frequency also reduced in the dark, but their small amplitudes slightly increased, while latency and clustering remained unchanged. First and second saccade frequencies were 75.3(4.5) % and 20.3(4.1) %; without visual fixation they dropped to 32.2(5.0) % and 3.8(1.2) %. The VOR gain was affected by vision only in unlesioned ears of UVD; gains for the horizontal-plane rose slightly, and the vertical-planes reduced slightly. All head-impulse compensatory saccades have a visual contribution, the magnitude of which depends on the symmetry of vestibular-function and saccade latency: BVL is more profoundly affected by vision than UVD, and second saccades more than first saccades. Saccades after UVD are probably triggered by contralateral vestibular function.

## Introduction

In health, the brain uses tonic vestibular activity to continuously stabilise gaze in anticipation of perturbation [1, 2]. This vestibulo-ocular reflex (VOR) operates with such fidelity that, even during a sudden, rapid, passive ‘head-impulse’ stimulus, gaze is smoothly maintained with an almost equal and opposite eye response [3]. When the ipsilateral VOR pathway is disrupted, a head-impulse stimulus generates an inadequate VOR such that gaze is pulled away from an earth-fixed visual target [4]. This gaze error provokes the brain to generate refixation saccades to compensate and re-acquire the gaze target [5–7]. The presence of a visible compensatory saccade was identified as an indirect clinical sign of an insufficient VOR [4], occurring at early latencies *during the head-impulse* (‘covert’ saccades) and later at normal visual saccade latencies (‘overt’ saccades) [5][4]. The delivery of an impulse in the plane of each semicircular canal (SCC) pair effectively (but incompletely) separates the individual SCC response [8], while the VOR and saccades effectively separate activity of the ear and brain respectively [9, 10] Thus, the quantitative head-impulse test represents the execution of a vestibular task and a visual correction.

What is the origin of these corrective saccades? Unlike the relatively simple VOR arc, many regions of the brain contribute to saccade characteristics, especially those involved with vision, attention, and movement [11], and thus show a more variable and more complex relationship with stimuli. For example, the reaction time (latency) of an individual saccade, as well as the distribution of many saccades [12], demonstrates that the saccadic decision is influenced by factors such as the task, cognitive state, and sensory modality [13, 14]; saccades to suddenly appearing visual targets usually occur within 200 ms, yet while ‘express’ saccades as early as ~100 ms that can be learned [15] are still at latencies which far exceed synaptic delays, indicating a computational decision-making process [16–18].

The vestibular system has a close relationship with the saccadic system [10]. Vestibular nuclei asymmetry, from visual and vestibular inputs [19], also triggers ‘quick-phase’ saccades in precisely the *opposite direction* of ongoing smooth VOR drift [20, 21], while head-impulse compensatory saccades are required in the *same direction* as the smooth VOR [4, 5, 22]. In complete darkness, to imaginary targets compensatory saccades were found to be reduced in frequency and amplitude after sub-total bilateral vestibular loss (BVL) [23]. While saccades were abolished in subjects with complete BVL when tested in the dark [24], this effect of vision was not complete for short-latency saccades after one subject with sub-total BVL, implying any residual vestibular input may also contribute [25]. Cervical proprioception has also been suggested as a potential trigger mechanism in BVL [25–27], but little high-velocity human data exists.

In the present study, we sought to better understand the sensory mechanisms that trigger compensatory saccades, what they compensate for, and whether there are differences in saccades generated in specific canal planes. To this end, we investigated the effect of peripheral vestibular function and visual input on compensatory saccade prevalence, amplitude, and latency in healthy and lesioned ears. We systemically examined these saccade parameters in response to head impulses in the planes of all semicircular canals, in normal controls, and after both unilateral and bilateral vestibular loss. The study also enabled us to compare first and subsequent saccades in their response to visual deprivation.

## Methods

### Participants

Sixteen adult human subjects without a history of visual loss, audio-vestibular symptoms, a known neurological disease, or recent neck or eye surgery, were recruited as the healthy normal control group (Table 1) from a subject pool previously published [12]. The presumed total unilateral vestibular deafferentation (UVD) group consisted of 22 subjects who underwent complete unilateral resection for vestibular schwannoma, confirmed through the complete loss of cervical and ocular vestibular evoked myogenic potentials (c/oVEMPS) to air- and bone-conducted stimuli [28], all tested more than six weeks after surgery when subjective visual horizontal (SVH) had returned to within the normal range (2 ° [29]) and spontaneous nystagmus in darkness was not visible. The bilateral vestibular loss (BVL) group consisted of eight subjects with severe isolated idiopathic BVL [30], all with a vHIT VOR gain >2SD below the normal range in all canal planes [12]. A single subject–previously reported [25]–was recruited with complete surgical bilateral vestibular deafferentation (BVD), after four separate surgeries for treatment of neuro-fibromatosis type 2 (NF2), who still had good vision and lived independently.

**Table 1:** The number of subjects of each case type, the number of canals tested and mean(SD) impulses for each subject, the number of subjects in each condition test order. UVDc = unilateral vestibular deafferentation contral-lesional, UVDi = unilateral vestibular deafferentation ispi-lesional, BVL = bilateral vestibular loss, BVD = bilateral vestibular deafferentation.

		Canal			Visual Condition Order	
Case	Subjects	Lateral	Anterior	Posterior	Light-Dark	Dark-Light
**Normal**	16	32, (17(8.9))	10, (7(6.5)	10, (12(10.6))	8	8
**UVDc**	22	22, (22(10.6))	21, (10(6.3))	21, (13(8.8))	14	8
**UVDi**	22	22, (32(8.7))	21, (17(9.5))	21, (22(10.2))	14	8
**BVL**	8	16, (26(9.0))	14, (17(11.6))	14, (21(11.8))	6	2
**BVD**	1	2, (41(13.9))	2, (17(13.6))	2, (25(7.7))	1	0

### Stimulus

The head impulse stimulus is a short duration (<200 ms), high acceleration (>2000 m/s^2^), high peak head velocity (100–300 °/s), small amplitude (~10–15°) head movement passively delivered with variable timing and direction [31, 32].

Head impulses were performed in the plane of each canal pair [8, 33]. Impulses plane sequence order was left and right lateral canals (horizontal), right anterior and left posterior canals (RALP), then left anterior and right posterior canals (LARP). Impulses were performed with a quasi-random side order, at variable peak head velocities and head amplitudes. The operator’s hands were placed firmly on the top of the subject’s head during horizontal plane impulses. The head was not re-centred after each impulse. Given that each impulse was of low amplitude (~10–15°) (Table 2), this allowed for delivery of two consecutive impulses in a given direction in order to make the stimulus unpredictable such that not all impulses were alternating direction e.g. left-right-left-right but could have been e.g. left-left-right-left.

**Table 2:** The number of head impulses and mean(SD) of stimulus characteristics for each canal in each visual condition.

Canal	Condition	Number	Amplitude (°)	Peak Velocity (°/s)	Peak Acceleration (°/s^2^)	Duration (ms)	Bounce (%)
**Lateral**	**Light**	2,968	13(3.8)	204(456)	3876(1015.4)	154(16.2)	33(16.6)
	**Dark**	2,846	14(3.8)	205(44.8)	3704(1004.2)	163(16.4)	31(15.8)
**Anterior**	**Light**	1,819	12(3.5)	174(38.8)	3418(1028.3)	157(24.8)	21(15.1)
	**Dark**	1,382	12(3.0)	174(35.9)	3418(1077.4)	159(21.5)	22(13.8)
**Posterior**	**Light**	2,023	11(3.5)	167(38.2)	3156(909.2)	152(26.2)	22(17.6)
	**Dark**	1,677	11(3.1)	163(37.5)	3004(987.7)	157(23.4)	22(16.0)

### Data capture

The experiment was conducted in two small adjoining rooms, both of which could be made completely dark by sliding doors, with light blocking strips and black curtains separating them. In all experiments the subject sat on a chair with their eyes 150 cm from a 2 cm diameter earth-fixed circular target on the wall positioned level with their eyes.

Three-dimensional head position and two-dimensional eye position was recorded at ~250 Hz by a video head impulse test (vHIT) system (USB goggles, Otometrics, Taastrup, Denmark). A laptop computer in the adjacent room captured data, including 120 fps video of the eye. Prior to calibration, goggle fit to the face, nose and scalp was ensured to be snug as possible. Immediately after calibration and just prior to data collection in each axis, low velocity head oscillations were performed within each plane to ensure eye tracking was low noise and properly calibrated. One right-handed operator delivered the stimulus while another controlled data collection in the adjacent room. The operator’s right-hand was placed on top of the head during RALP and LARP impulses, with care taken to avoid pulling on the hair, skin, or otherwise interfering with the goggles and elastic straps.

To facilitate the operator maintaining the subject’s correct gaze angle in light and darkness, the goggle software generated a head angle position that was aligned to ~40 ° either side of center, while gaze was maintained towards the central target. This provided real-time monitoring of head and eye position to the operator, who relayed any required corrections throughout the test.

Two lighting conditions were used: with the rooms lit by eight 60W incandescent light bulbs (*light*, or ‘with fixation’) or the rooms completely darkened by all lights switched off and the doors closed (*dark*, or ‘without fixation’) to prevent any light leaking (the camera recorded only invisible infrared light). Prior to any test recording, all subjects were shown the target and instructed for each test condition. During ‘with visual fixation’ test periods, subjects were instructed to fixate on the target throughout the test. During ‘without visual fixation’ test periods, subjects were instructed to fixate on the imagined location of the same target. Subjects of each group were pseudo-randomly tested in one of two visual condition orders; light then dark, or dark then light.

Test recordings were performed at least 30 days elapsed since any other previous head-impulses e.g. clinical examinations, to help minimize any potential learning. Some subjects were not included in the study when the pupil size in darkness meant unreliable pupil tracking due to eye lid and lashes artefact. During the experiment all subjects were well, attentive, and reported no visual problems (besides small refractive errors). Head impulses were collected within 3–4 minutes per canal pair, which all subjects tolerated and maintained alertness throughout.

### Data analysis

Head and eye velocity data were processed offline using custom software (LabView v2012, National Instruments, Austin, TX, US). Traces were processed concurrently with the high-speed video to remove traces with artifacts from blinks, half-blinks, and other tracking errors [34]. Head impulses were detected with a velocity profile described before [35]. Head impulse onset is defined by the time axis intercept of the line tangential to the head velocity at the peak acceleration. Laplacian of Gaussian filters are used to measure gradients to reduce the sensitivity to noise. Excessive, incomplete, or otherwise outlying head velocity traces were visually identified for each subject’s canal and discarded manually.

The VOR gains were calculated using a previously reported robust ratio method of comparing impulsive head and eye movements using the cumulative head and eye velocity ratio from 60 ms prior to peak acceleration to zero crossing [35]. That is, a position ratio.

We separated the saccades from the VOR using a regularization method that exploits their differential properties, i.e. the smoothness of the VOR and the temporal characteristics of saccades. First saccades were localized by selecting zero crossing times in the eye acceleration signal that exhibit a large jerk magnitude (i.e. sharp crests and valleys), using a method previously described [12]. Each saccade was then modelled as the sum of two Gaussian functions (${\hat{v}}_{\text{sac}}$) which is designed to be representative of saccadic signals typically found in vHIT, which included post-saccadic oscillations of the iris typically recorded in video methods that track the pupil [36–38]. The parameters *A*, *B*, *t*_*peak*_ and σ were defined as those that minimize the cost function *J* and were found using iterative gradient descent. *J* is designed such that only high frequency components of the error signal are minimized (as mediated by high-pass filter *f*_*HP*_); thereby preserving the residual low frequency slow phase signal. Saccade attributes amplitude (°), peak velocity (°/s), and onset latency (ms) could then be determined numerically from the ${\hat{v}}_{\text{sac}}$ equation.

$$\hat{v_{sac}}(t)=Ae^{-\frac{{\left(t-t_{peak}\right)}^{2}}{2\sigma^{2}}}-Be^{-\frac{{\left(\left\{t-t_{peak}-2\sigma \right)\right)}^{2}}{2\sigma^{2}}}$$

$$J=\int {\left(f_{HP}*(v_{eye}-\hat{v_{sac}})\right)}^{2}dt$$

Saccades were detected and fit automatically, however where the solution was incorrect due to ambiguity in the data, the parameters were optimized by manual intervention to subjectively maintain a consistent residual VOR with reference to the head motion.

Only saccades after 50ms were included in this analysis since earlier micro-saccades are probably due to the visual fixation task [39]. Unless otherwise noted, all saccade characteristics reported in this study were limited to the compensatory “catch-up” direction i.e. eye movement in the same direction as the smooth VOR and opposite to the head movement. The grand total of clean traces was 12,715 (28(8) impulses/canal/subject), that included 6,561 first, 3,097 second, and 813 third compensatory saccades.

The visual error prior to each saccade was also calculated. Integration to determine the position signal introduces a positional drift due to several sources of error e.g. sample noise, goggle slip, sensor velocity drift, calibration, and requires the assumption that gaze is on the target prior to and following the head impulse, as maintained by compensatory eye movements. Nevertheless, a positional signal was approximated by assuming a proportion of sensor drift and eye signal scaling, which was normalized to provide a consistent visual error signal correlated in time at the onset of each saccade.

### Statistical analysis

Statistical analysis was performed using statistical analysis software R (v3.5.1) [40] and packages [40–48]. Due to the nature of the head-impulse test and data cleaning process, subjects did not have the same number of head-impulses and saccades. Therefore, analyses within the framework of generalized estimating equations (GEE) assuming exchangeable correlation, was used to investigate main and interaction effects on *dependent variables*: for saccade amplitude, onset latency, latency cluster, and VOR gain we used linear regression, while for saccade frequency we used binomial logistic regression probability (expressed as a percentage (%)). Saccade clustering was determined using standard deviation (SD) of the onset latency for each canal in each condition. GEE accounts for the correlation in observations due to having multiple observations per subject and provides a robust population level estimate [49]. Comparison significance was set to 0.05 (95% CI).

Post-hoc analyses were based on paired comparisons of estimated marginal means (EMM), unless otherwise stated. The pseudo coefficient of determination (R^2^) between two continuous variables was determined from generalized linear mixed models (GLMM), with random intercepts for subjects [50, 51]. The conditional r^2^ provides a goodness of fit based on fixed and random effects while marginal r^2^ provides a goodness of fit based on fixed effects only [52]. To better isolate the effect of vision in the models of saccade characteristics and VOR gain, the y-intercept was measured at a VOR gain of 1.0.

Of the lesion groups with more than one subject (Normal, UVD and BVL), the ears of UVD subjects were separated into the normal contralesional (UVD_contra_) and abnormal ipsilesional (UVD_ipsi_). Thus, the *independent variables* for saccade data were the case groups (Normal, UVD_contra_, UVD_ipsi_, BVL), the lighting conditions (Light, Dark), condition test order (Light-Dark, Dark-Light), and the saccade Sequence (First, Second, Third). For VOR gain analysis, only case groups and lighting conditions were used, as well as peak head velocity that was split into Low (120–200 °/s) and High (200–300 °/s) groups.

### Study ethics

This study was carried out with approval by the Sydney Local Health District—Royal Prince Alfred Zone (protocol number: X13-0425) Human Research Ethics Committee. All subjects freely gave written informed consent in accordance with the Declaration of Helsinki and later amendments.

## Results

**Fig 1:**
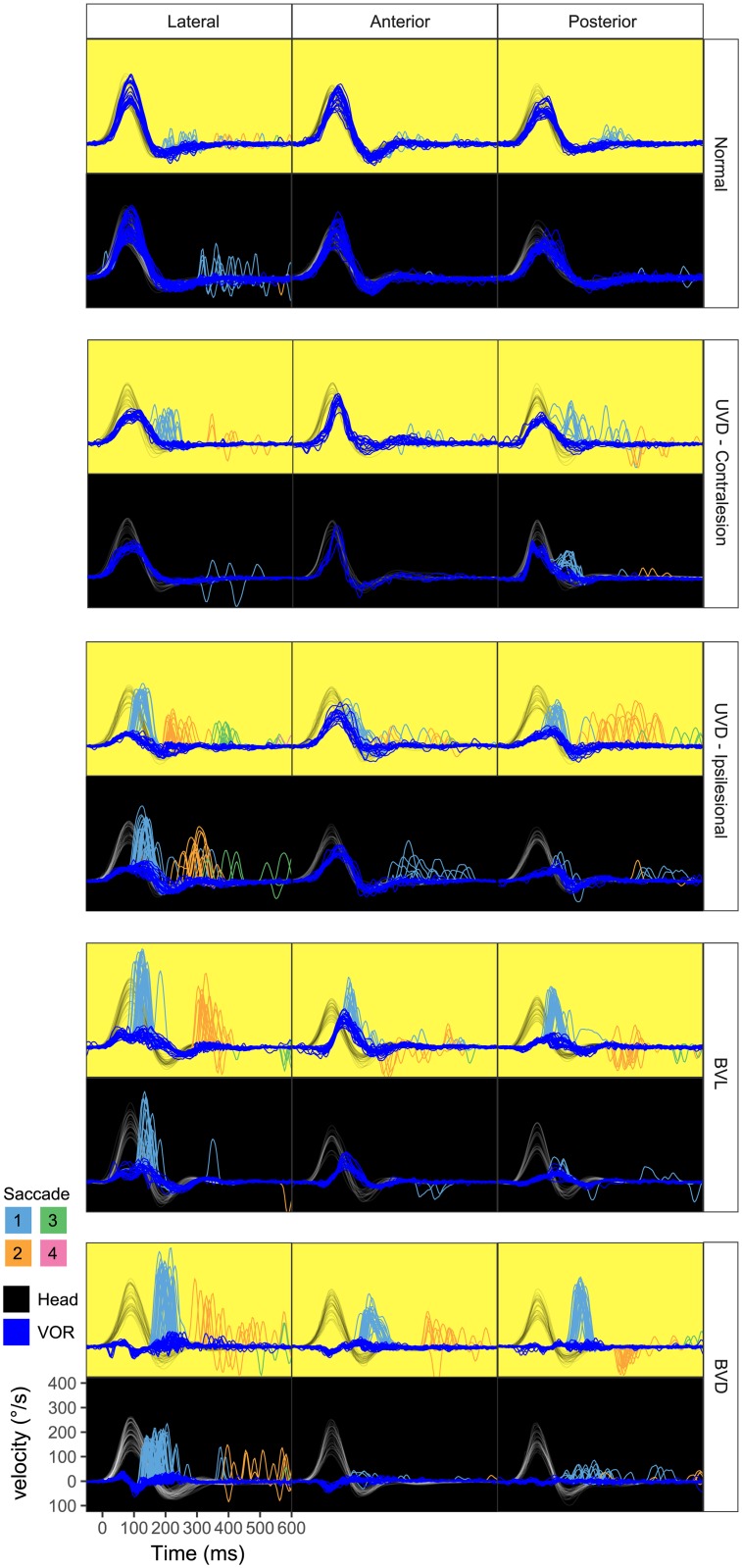
Examples of head and eye velocity traces for each case group in each canal of the right ear. Traces with a yellow background were recorded in the light with a visual target while those with a dark background were recorded in darkness while subjects imagined the same target. For Normal ears, without visual fixation, saccades became less frequent but later and larger in amplitude. For UVD_contra_ ears, saccades also became later and less frequent. For UVD_ipsi_ ears, saccade amplitudes became smaller in the posterior canal (PC) only, but remained unchanged in frequency and latency in the lateral canal (LC) and anterior canal (AC), while the second saccades became later, smaller in amplitude, and less clustered. For BVL, in the dark, the first saccade showed a reduction in amplitude and frequency, while the second saccades were virtually absent. For BVD, saccade amplitudes were dramatically reduced for all canals, while their average latency increased, and clustering decreased. UVDc = unilateral vestibular deafferention contralesion, UVDi = unilateral vestibular deafferention ipsilesion, BVL = bilateral vestibular loss.

**Fig 2:**
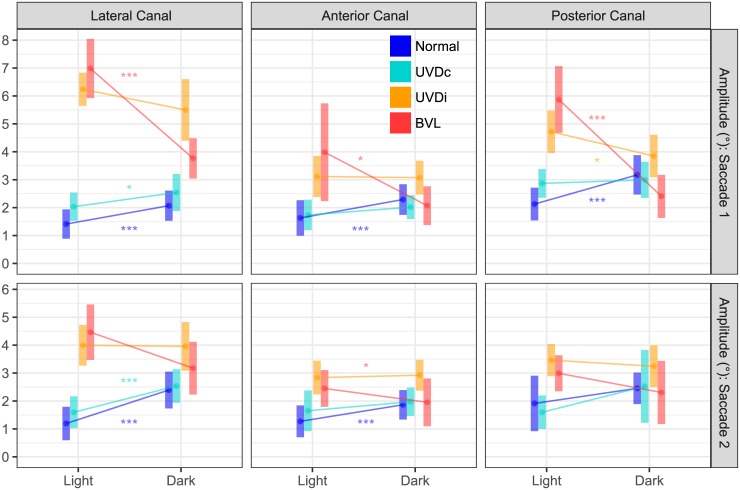
The effect of visual condition on saccade amplitudes of healthy and lesioned ears. When visual fixation is removed (dark) the first saccade amplitude of the BVL group reduced in all canal planes. In contrast, the affected ear of UVD (UVD_ipsi_) did not demonstrate as great a saccade amplitude reduction. The second saccade showed less dramatic effects than the first. Saccades from unlesioned ears (Normal, UVD_contra_) became slightly larger in dark. UVDc = unilateral vestibular deafferention contralesion, UVDi = unilateral vestibular deafferention ipsilesion, BVL = bilateral vestibular loss. The bars represent the estimated marginal mean(CI). Asterisks indicate a significant difference between visual conditions (*: p < 0.05, **: p < 0.01, ***: p < 0.0001).

**Fig 3:**
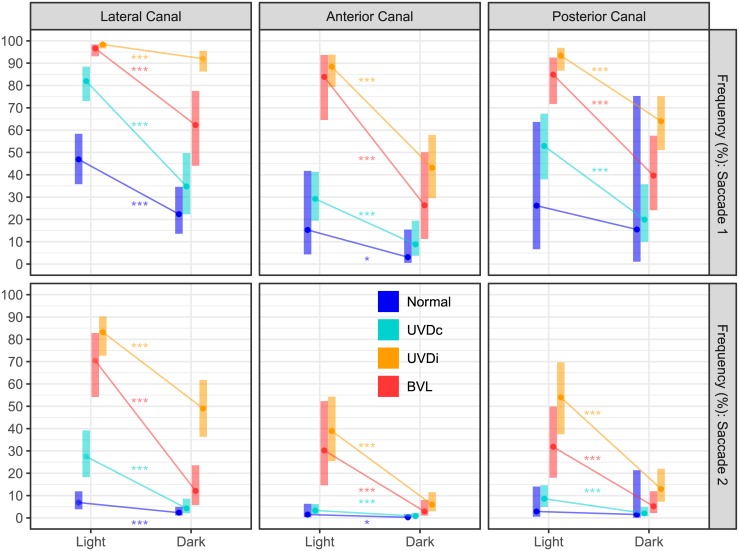
The effect of visual condition on saccade frequency in healthy and lesioned ears. The estimated marginal mean(CI) of the probability of a saccade, represented here as frequency, shows that all case groups were affected by the lack of visual fixation. However, for the first and second saccade of the lateral canal (LC), UVD_ipsi_ was less affected than BVL, but was usually similarly affected in the anterior and posterior canals (AC, PC). UVDc = unilateral vestibular deafferention contralesion, UVDi = unilateral vestibular deafferention ipsilesion, BVL = bilateral vestibular loss. Asterisks indicate a significant difference between visual conditions (*: p < 0.05, **: p < 0.01, ***: p < 0.0001).

**Fig 4:**
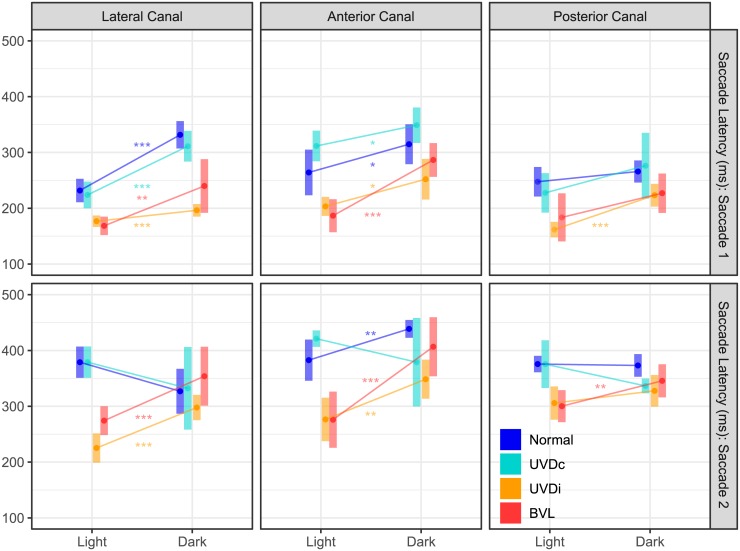
The effect of visual condition on saccade latency on healthy and lesioned ears. The estimated marginal mean(CI) of saccade latency shows a prolonged onset without visual fixation in most cases groups. Lesioned ears (BVL, UVD_ipsi_) were at a similar latency with visual fixation, earlier than unlesioned ears (Normal, UVD_contra_), and were delayed similarly without visual fixation. UVDc = unilateral vestibular deafferention contralesion, UVDi = unilateral vestibular deafferention ipsilesion, BVL = bilateral vestibular loss. Asterisks indicate a significant difference between visual conditions (*: p < 0.05, **: p < 0.01, ***: p < 0.0001).

**Fig 5:**
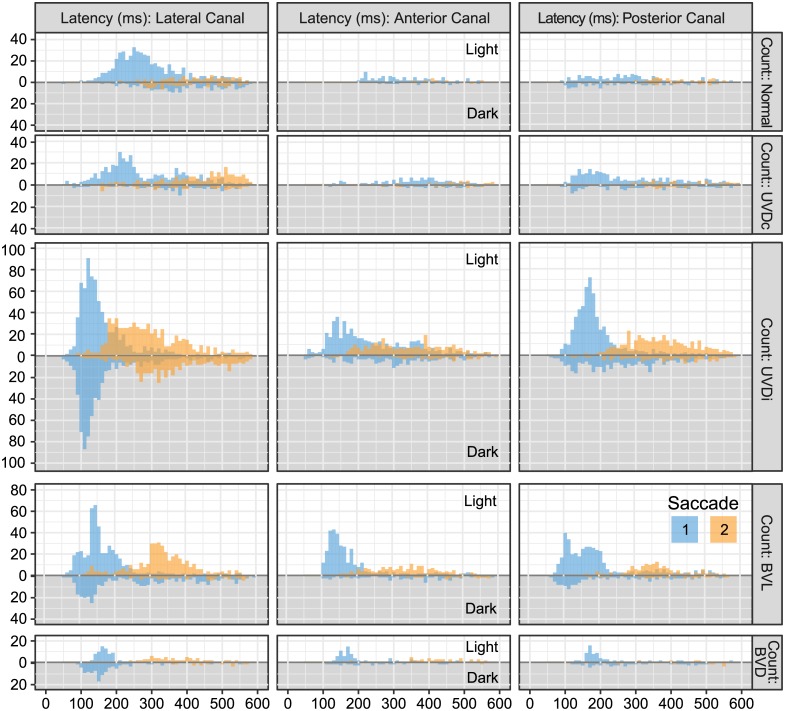
The influence of visual fixation on the number of saccades: First saccades are affected to a lesser degree than the second saccade. The white background shows the number of saccades observed with visual fixation, while the dark background shows the number of saccades without visual fixation. In lesioned ears (UVD_ipsi_, BVL, BVD) the number of first and second saccades decreases in the dark. Since the number of impulses recorded from the vertical (AC, PC) and lateral canals (LC) were dissimilar (Table 1), the findings for these two groups should not be compared. UVDc = unilateral vestibular deafferention contralesion, UVDi = unilateral vestibular deafferention ipsilesion, BVL = bilateral vestibular loss, BVD = bilateral vestibular deafferentation.

**Fig 6:**
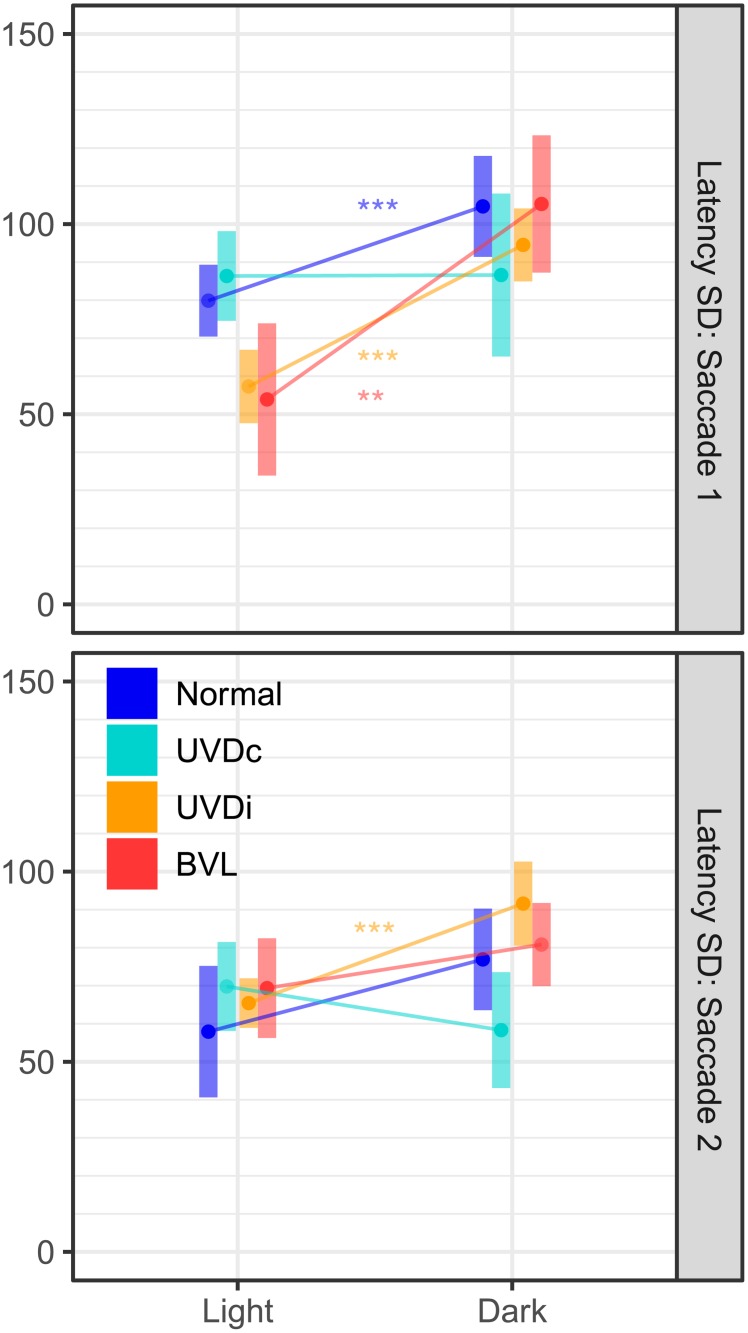
The effect of visual condition on saccade clustering in healthy and lesioned ears. The estimated marginal mean(CI) of saccade onset latency clustering, calculated using standard deviation, shows that the first saccade of lesioned ears (UVD_ipsi_, BVL) were more clustered than unlesioned ears (Normal, UVD_contra_). However, without visual fixation the clustering was similar for all case groups. UVDc = unilateral vestibular deafferention contralesion, UVDi = unilateral vestibular deafferention ipsilesion, BVL = bilateral vestibular loss. Asterisks indicate a significant difference between visual conditions (*: p < 0.05, **: p < 0.01, ***: p < 0.0001).

Fig 1 illustrates the effects of visual fixation on refixation saccades of lesioned and unlesioned ears of single subjects. As also illustrated in Figs 2–6, only minor changes were evident in the small saccades recorded from unlesioned ears where saccade frequency decreased slightly, and amplitude increased slightly as visual fixation was removed. Unilaterally deafferented ears also demonstrated only modest effects of visual deprivation, with a reduced saccade frequency and increased latency for all canals and reduced saccade amplitude for the PC only. In contrast, the bilaterally lesioned ears (BVL) demonstrated a decrease in the saccade frequency and amplitude as well as an increase in latency and dispersion. In complete bilateral vestibular deafferentation (BVD), when visual fixation was removed, saccades reduced in frequency but were not completely abolished, their amplitudes decreased dramatically, and latencies became more variable. Subjects with bilateral vestibular loss (BVL) demonstrated more dramatic effects of vision on all saccade metrics (frequency, amplitude, latency) compared to the affected ear of unilateral vestibular loss (UVDipsi).

### 1.1 Normal controls

**Fig 7:**
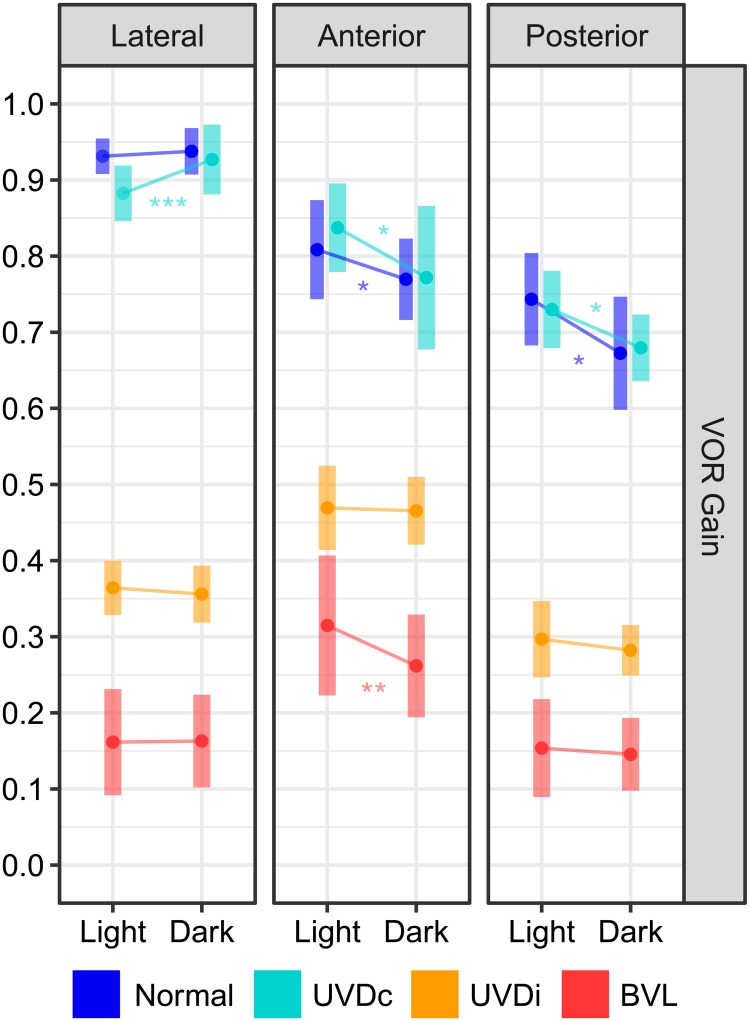
The effect of visual fixation on VOR gain. The estimated marginal mean(CI) of ears from Normal and UVD_contra_ ears showed small changes, but lesioned ears (UVD_ipsi_, BVL) were unaffected. UVDc = unilateral vestibular deafferention contralesion, UVDi = unilateral vestibular deafferention ipsilesion, BVL = bilateral vestibular loss. Asterisks indicate a significant difference between visual conditions (*: p < 0.05, **: p < 0.01, ***: p < 0.0001).

In normal subjects, amplitudes of the first saccade were minuscule with fixation (LC: 1.4(0.27)°; AC: 1.6(0.32)°; PC: 2.1(0.30)°) and were slightly larger without fixation dfs = 6; F-ratio = 4.72; p < 0.0001) (Fig 2, Table 3). Saccade frequency reduced in the dark (dfs = 3; F-ratio = 22.6; p < 0.001). When visual fixation was removed, saccades from the LC and AC were delayed, but saccades from the PC were unchanged (Figs 4 and 5, Table 3, dfs = 6; F-ratio = 8.0; p < 0.0001). Saccades were less clustered when vision was denied (dfs = 1; F-ratio = 29.8; p < 0.001, Fig 6, Table 3). The VOR gains were 0.93(0.01), 0.81(0.03), 0.74(0.3) for the LC, AC, and PC. A significant three-way analysis (dfs = 6; F-ratio = 3.748; p = 0.001) revealed that although the LC remained unaffected by the lack of vision (p = 0.612), both vertical canal gains reduced slightly (p < 0.038) (Fig 7).

**Table 3:** Saccade characteristics in light and dark. The values are the estimated marginal mean and standard error of the mean(SEM), while the contrast provides the p-value comparison between light and dark conditions. Values are based on four-way interaction models. Clustering refers to the unitless standard deviation (SD) of the latency for each saccade from each canal type. UVDc = unilateral vestibular loss, contralesional; UVDi = unilateral vestibular loss, ipsilesional; BVL = bilateral vestibular loss.

			First			Second		
Parameter	Case	Condition	Lateral	Anterior	Posterior	Lateral	Anterior	Posterior
**Frequency (%)**	**Normal**	**Light**	46.9±5.85	15.3±9.11	26.1±15.76	6.9±1.97	1.5±1.12	2.9±2.43
		**Dark**	22.4±5.38	3.1±2.67	15.5±18.76	2.4±0.89	0.3±0.25	1.5±2.18
		**Contrast**	*< 0*.*001*	0.134	0.379	*0*.*001*	0.226	0.381
	**UVDc**	**Light**	82±3.91	29.2±5.65	52.9±7.73	27.5±5.4	3.3±1.06	8.6±2.41
		**Dark**	34.8±7.15	8.8±3.74	19.9±6.55	4.3±1.56	0.8±0.42	2±0.92
		**Contrast**	*< 0*.*001*	*< 0*.*001*	*< 0*.*001*	*< 0*.*001*	*0*.*002*	*0*.*001*
	**UVDi**	**Light**	98.3±0.61	88.4±3.58	93.4±2.44	83.2±4.43	38.9±7.57	54±8.52
		**Dark**	92±2.28	43.1±7.42	64±6.26	49±6.64	6±2.06	12.9±3.7
		**Contrast**	*0*.*001*	*< 0*.*001*	*< 0*.*001*	*< 0*.*001*	*< 0*.*001*	*< 0*.*001*
	**BVL**	**Light**	96.6±1.26	83.8±7.24	84.9±5.20	70.5±7.46	30.2±9.99	31.9±8.37
		**Dark**	62.3±8.86	26.3±10.22	39.6±8.84	12.1±4.38	2.9±1.54	5.2±2.27
		**Contrast**	*< 0*.*001*	*< 0*.*001*	*< 0*.*001*	*< 0*.*001*	*0*.*004*	*< 0*.*001*
**Amplitude (°)**	**Normal**	**Light**	1.4±0.27	1.6±0.32	2.1±0.3	1.2±0.31	1.3±0.29	1.9±0.51
		**Dark**	2.1±0.28	2.3±0.28	3.2±0.36	2.4±0.34	1.9±0.27	2.5±0.29
		**Contrast**	*< 0*.*001*	*< 0*.*001*	*< 0*.*001*	*< 0*.*001*	*< 0*.*001*	0.204
	**UVDc**	**Light**	2.0±0.26	1.7±0.28	2.9±0.26	1.6±0.29	1.6±0.37	1.6±0.31
		**Dark**	2.5±0.34	2.0±0.22	3.0±0.33	2.5±0.31	2.0±0.26	2.5±0.67
		**Contrast**	*0*.*019*	0.149	0.632	*< 0*.*001*	0.44	0.106
	**UVDi**	**Light**	6.2±0.30	3.1±0.37	4.7±0.39	4.0±0.37	2.8±0.31	3.5±0.29
		**Dark**	5.5±0.56	3.1±0.31	3.8±0.39	4.0±0.44	2.9±0.28	3.2±0.38
		**Contrast**	0.128	0.892	*0*.*008*	0.881	0.683	0.525
	**BVL**	**Light**	7.0±0.54	4.0±0.89	5.9±0.61	4.5±0.51	2.5±0.34	3.0±0.33
		**Dark**	3.8±0.37	2.1±0.35	2.4±0.39	3.2±0.48	2±0.44	2.3±0.58
		**Contrast**	*< 0*.*001*	*0*.*033*	*< 0*.*001*	0.091	*0*.*03*	0.155
**Latency (ms)**	**Normal**	**Light**	232±10.7	264±20.9	248±13.5	379±14.3	383±18.8	376±7.5
		**Dark**	332±12.5	315±18.2	266±10.1	327±20.5	439±8.1	373±10.3
		**Contrast**	*< 0*.*001*	*0*.*036*	0.204	0.055	*0*.*002*	0.602
	**UVDc**	**Light**	224±12.2	312±14.0	228±18.0	379±14.3	421±7.5	376±21.8
		**Dark**	311±14.0	349±16.2	276±30	332±37.7	379±40.4	336±6.7
		**Contrast**	*< 0*.*001*	*0*.*042*	0.079	0.221	0.31	0.057
	**UVDi**	**Light**	177±5.2	203±8.7	162±7.1	225±13.4	276±19.8	306±15.2
		**Dark**	196±5.7	252±18.6	223±10.4	298±11.5	348±17.9	328±14.5
		**Contrast**	*0*.*001*	*0*.*023*	*< 0*.*001*	*< 0*.*001*	*0*.*003*	0.224
	**BVL**	**Light**	168±8.4	187±15.1	184±22	274±13.2	276±25.6	300±14.6
		**Dark**	240±24.5	287±15.4	227±18	354±26.9	407±27	346±15.1
		**Contrast**	*0*.*003*	*< 0*.*001*	0.178	*0*.*006*	*< 0*.*001*	*0*.*001*
**Clustering**	**Normal**	**Light**	77±4.8	81±6.2	82±5.1	55±9	59±9.5	60±8.9
		**Dark**	102±6.8	106±7.5	107±7.3	74±6.7	78±7.7	79±7.2
		**Contrast**	*0*.*001*	*0*.*001*	*0*.*001*	0.083	0.083	0.083
	**UVDc**	**Light**	83±5.8	87±7.1	88±6.5	67±6.2	71±7	72±6.1
		**Dark**	84±11.2	88±11.2	89±11.1	55±8.0	59±8.2	60±8.2
		**Contrast**	0.982	0.982	0.982	0.205	0.205	0.205
	**UVDi**	**Light**	54±5.0	58±6.2	59±5.2	62±4.0	66±4.7	67±3.7
		**Dark**	92±5.2	96±6.2	96±5.0	88±6.0	93±7.0	94±5.3
		**Contrast**	*< 0*.*001*	*< 0*.*001*	*< 0*.*001*	*< 0*.*001*	*< 0*.*001*	*< 0*.*001*
	**BVL**	**Light**	51±10.4	55±11.2	56±9.9	66±7.5	70±7.8	71±6.0
		**Dark**	102±9.4	106±9.2	107±9.9	78±6.4	82±6.2	83±5.6
		**Contrast**	*0*.*004*	*0*.*004*	*0*.*004*	0.058	0.058	0.058

### 1.2 Healthy ears of UVD subjects

In healthy UVD ears, the first saccade amplitudes were small (LC: 2.0(0.26) °; AC: 1.7(0.28) °; PC: 2.9(0.26) °). Only the LC saccades increased in amplitude without fixation (dfs = 6; F-ratio = 4.72; p = 0.0001; Fig 2, Table 3). In the dark, saccades were less common from all canals (dfs = 3; F-ratio = 6.21; p = 0.0003, Fig 3, Table 3). Saccade latency from the LC and AC increased without visual fixation but the PC was unchanged (dfs = 6; F-ratio = 8.0; p < 0.001; Fig 4, Table 3). Saccade clustering was unaffected by vision (dfs = 1; F-ratio = 29.8; p = 0.928) (Table 3).

The VOR gain (LC: 0.87(0.02); AC: 0.84(0.03); PC: 0.73(0.03)) was affected by the lack of vision, (dfs = 6; F-ratio = 3.748; p = 0.001) rising slightly (0.05) for the horizontal canal plane of UVD and reducing slightly for both vertical canals (AC: 0.08; PC: 0.05) (Fig 7).

### 1.3 UVD ears

For lesioned UVD ears, when vision was present, saccade amplitudes were 2.5–4 fold larger (dfs = 6; F-ratio = 4.72; p < 0.0001) than in healthy ears (LC: 6.2(0.30) °; AC: 3.1(0.31) °; PC: 4.7(0.39) °) (Fig 2, Table 3). In the dark, LC and AC saccade amplitudes remained unchanged; only saccades from the PC became smaller (p = 0.008). Saccades from all canals were less common in the absence of visual fixation (dfs = 3; F-ratio = 22.6; p = 0.003) (Fig 3, Table 3). The LC was the least affected and reduced only by 17.6%, while the % reduction of saccade frequency for the vertical canals was more than double that of the LC. In UVD ears, first refixation saccade latencies from the LC occurred earlier than normal ears (dfs = 6; F-ratio = 8.0; p < 0.001). Without visual fixation, latencies for the LC were only slightly increased (+19.6 ms; p < 0.0001), while the vertical canals increased by more than double the LC (p < 0.023) (Table 3, Fig 4). Saccade clustering was significantly affected by vision, with an increase of saccade dispersal in the dark (dfs = 1; F-ratio = 29.8; p < 0.001).

The VOR gains were 0.36(0.02), 0.47(0.02), 0.25(0.02) from the LC, AC, and PC with fixation (Fig 7). All were unaffected by vision (dfs = 6; F-ratio = 3.748; p = 0.093).

### 1.4 Bilateral vestibular loss

For BVL ears, when visual fixation was present, saccade amplitudes from all canals were 2.5–5 fold larger than from healthy ears (LC: 7.0(0.54); AC: 4.0(0.89); PC: 5.9(0.61); dfs = 6; F-ratio = 4.72; p = 0.008) (Fig 2, Table 3). Saccade amplitudes from all canals were dramatically reduced, halving when vision was denied (LC: 3.8(0.37); AC: 2.1(0.35); PC: 2.4(0.39); p = 0.033).

With visual fixation, saccade frequency was similar to lesioned UVD ears LC: 97(1.3) %; AC: 84(7.2) %; PC: 85(5.2) %; p > 0.063) and in darkness reduced dramatically from all three canals (LC: -57%, AC: -51%, PC: -46%; dfs = 3; F-ratio = 22.6; p < 0.0001; Fig 3). With visual fixation, first saccade latency for all canal planes was similar to the lesioned ear of UVD (p > 0.348) and increased by similar amount in the dark (p > 0.09) (Fig 4, Table 3). Saccade clustering also decreased in the dark (dfs = 1; F-ratio = 29.8; p = 0.0034) to be similar to the lesioned ear of UVD (p = 0.999).

The VOR gains for each canal in light was LC: 0.16(0.04), AC: 0.31(0.05), and PC: 0.15(0.03) (Fig 7). Only the AC decreased slightly (-0.06) without fixation dfs = 6; F-ratio = 3.748; p = 0.0032), as the others were unchanged.

In the single subject with complete BVD, first saccade amplitudes from the LC were 7.3(SD 2.62) ° with visual fixation and almost halved to 3.1(0.94) ° without visual fixation, while the AC and PC plane saccades reduced even further (Fig 1, Table 4). Saccade frequency also reduced when visual fixation was removed (LC: -10%; AC 56%; PC: 20%), but surprisingly saccades were not completely eliminated. Saccade latencies with and without vision in the LC were 165(SD 17) ms and 168(SD 70), with dispersal increasing from 17(SD 1.2) to 66(SD 13), with the AC and PC planes following a similar but more pronounced changes. The VOR gain values were already very low and changed by less than 0.02(SD 0.02) in the dark.

**Table 4:** The mean(SD) of saccade and the VOR gain characteristics in one subject with complete BVD. Canals of the same type are combined from both ears. NA indicates an insufficient number of saccades to determine metric. Clustering refers to the standard deviation of saccade latency.

		First Saccade			Second Saccade		
Parameter	Condition	Lateral	Anterior	Posterior	Lateral	Anterior	Posterior
**Frequency (%)**	**Light**	100±0	98±2	100±0	96±5	64±22	65±39
	**Dark**	90±7	42±1	80±25	28±0	4±NA	18±7
**Amplitude (°)**	**Light**	7.3±2.62	6.6±2.35	6.7±2.06	2.3±1.17	1.5±0.97	0.6±0.24
	**Dark**	3.1±0.94	1.1±0.89	1.2±0.7	1.7±0.63	0.6±NA	0.7±0.33
**Latency (ms)**	**Light**	165±17	190±67.4	205±79.6	362±70.7	424±79.2	414±76.6
	**Dark**	168±70.2	236±102.3	281±118.6	472±68.9	547±NA	477±112.7
**Clustering**	**Light**	17±1.2	51±39.8	60±47	69±10.1	72±7.2	39±NA
	**Dark**	66±13.3	104±16.7	96±18.7	69±0	NA	122±NA
**VOR Gain**	**Light**	-0.03±0.026	0.00±0.082	-0.01±0.029			
	**Dark**	-0.01±0.024	-0.01±0.073	-0.01±0.031			

### 1.5 How do individual canals differ in their saccade metrics?

A significant three-way interaction (dfs = 6; F-ratio = 4.72; p = 0.0001) found that in the lesioned ears (UVD_ipsi_, BVL), regardless of visual condition, the first saccades of LC impulses showed the largest amplitudes (p < 0.02), while AC impulses were not different (p < 0.052) (Fig 2). For unlesioned ears (normal, UVD_contra_), the PC impulses demonstrated a trend to produce the largest saccade amplitudes (p < 0.055). Overall, compensatory saccades were most frequent in LC impulses, followed by the PC, then the AC (p < 0.001) (Fig 3, Table 3). The latency of saccades was typically earliest in the LC, PC, then AC, with the mean difference across case groups -3(39.2) ms in light and +14(44.3) ms in darkness.

### 1.6 How do early and late saccades differ?

#### Covert and overt saccades

From all case groups and in both visual conditions saccades that occurred during the head impulse (“covert”) saccade amplitudes were always larger in amplitude than saccades that occurred after the head impulse (“overt”) (dfs = 1; F-ratio = 81.94; p < 0.001).

#### First and second saccades

In normal controls, when there was visual fixation, we found no difference in the amplitude of the first and second saccade (dfs = 3; F-ratio = 16.97; p > 0.13).

With visual fixation, all canals of BVL and the LC and PC of both lesioned and unlesioned UVD ears had significantly larger first saccades compared with second saccades (dfs = 1; F-ratio = 151.68; p < 0.03).

We examined the influence of saccade number on the effect of vision for each case group by a three-way comparison (dfs = 3, F = 17.99, p < 0.0001). For normal subjects, the effect of vision on the saccade frequency was mean(SEM) 12.7(4.86) % *smaller* (p = 0.0092) and on latency was 62(30.62) ms *less* (p = 0.0413) for the second saccade compared with the first, while the effect on amplitude and clustering was similar (p > 0.35). For unlesioned UVD ears, the effect on latency was 73(26.40) ms *less* (p = 0.0055) and on clustering was 49.0(21.79) *less* (p = 0.0247) for the second saccade, while frequency and amplitude were similar (p > 0.061). For lesioned UVD ears, the effect of vision on frequency was 27.5(7.82) % more (p = 0.004) and on amplitude was 1.1(0.47) ° *more* (p = 0.0193) for the second saccade, while latency and clustering were similar (p > 0.077). For BVL subjects, the effect of vision on frequency was 36.1(6.00) % *more* (p < 0.0001), on amplitude was 1.8(0.314) ° *less* (p < 0.0001), and on clustering was 44.5(15.60) *less* (p = 0.0043) for the second saccade, while the effect on latency was similar (p = 0.82).

### 1.7 Are anti-compensatory saccades also affected by vision?

Anti-compensatory saccades occurred slightly more often when the order of the visual condition was light-dark compared to dark-light (Fig 1) (+3.9(1.70) %, dfs = 1; F-ratio = 0.2; p = 0.037). The frequency of compensatory saccades was unaffected by the condition order (p = 0.5).

### 1.8 Does vision affect the VOR gain?

As shown as Fig 7 and described earlier for each case group, only minor changes in the VOR gain were observed between visual conditions. Significant factors (p < 0.047) affecting the VOR gain included the case group (dfs = 3; F-ratio = 494.5), the canal group (df = 2; F-ratio = 47.55), and the visual condition (df = 1; F-ratio = 3.9). Subject age group (df = 2; F-ratio = 1.79), sex (df = 1; F-ratio = 0.66), and the condition test order (df = 1; F-ratio = 0.106) was not significant (p > 0.17).

### 1.9 Are there differences in abducting and adducting saccade parameters?

Adducting and abducting horizontal saccades of the right eye, to right UVD and left UVD lesions respectively, were of similar amplitude (df = 1; F-ratio = 0.066), frequency (df = 1; F-ratio = 1.47), and latency (df = 1; F-ratio = 2.454) in and between visual conditions (p > 0.11). The VOR gain was likewise unaffected (dfs = 2; F-ratio = 0.164; p = 0.8483).

### 1.10 The relationship between VOR gain on saccade metrics

#### Amplitude

**Fig 8:**
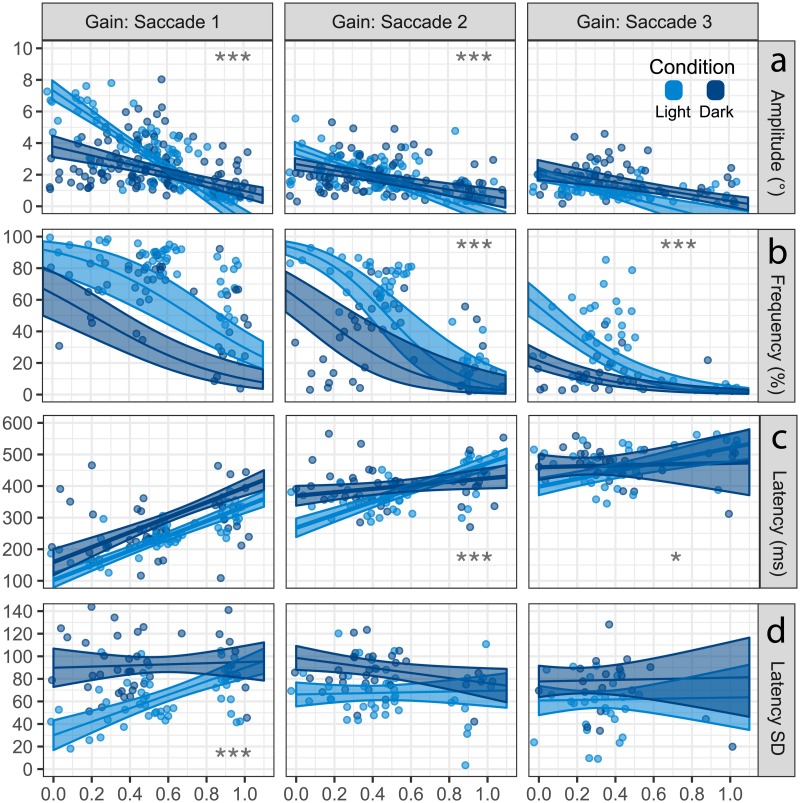
Saccade characteristics varied as a function of the VOR gain and visual condition. The colored lines show the estimated marginal mean(SEM) for each visual condition and individual points represent the mean of each individual subject’s canals. A) The first saccade amplitudes became increasingly affected by the loss of visual fixation as the VOR gain reduced, however, later saccades were less affected. B.) All saccades were less common without visual fixation, especially as the VOR gain reduced. The ribbon is non-linear due to calculation using binomial logistic regression. C.) The first saccade latency became earlier with decreasing VOR gain and was delayed without visual fixation. Latency of the second and third saccades were less affected by the reduction in VOR gain and the loss of visual fixation. D.) Clustering of the first saccade increased as the VOR gain reduced, however, without visual fixation clustering decreased and showed little relationship with the VOR gain. Asterisks indicate a significant difference in the slope between visual conditions (*: p < 0.05, **: p < 0.01, ***: p < 0.0001).

Saccade amplitude showed a strongly correlated negative relationship (conditional r^2^ = -0.80, marginal r^2^ = -0.17) with the VOR gain (Fig 8): saccade amplitudes increased as VOR gain reduced, with a significant interaction of visual condition and saccade number (p < 0.0001). Comparing visual conditions, all saccades showed a higher intercept at 1.0 without visual fixation (p < 0.001), but only the first and second saccade showed a steeper slope in the light compared with the dark (p < 0.001). Comparing saccade numbers, with visual fixation all saccades showed a similar intercept at 1.0 (p > 0.165), while the first saccade showed a steeper slope than the second and third saccade (p < 0.0001). Without visual fixation the intercept of first saccade was more than the third saccades (p < 0.0254), and all saccades showed a similar slope (p > 0.100).

#### Frequency

Saccade frequency also showed a negative relationship (conditional r^2^ = -0.69, marginal r^2^ = -0.61) with the VOR gain (Fig 8); saccade frequency increased as the VOR gain reduced, with a significant interaction of visual condition and saccade number (p = 0.0165). Comparing visual conditions, all saccades showed a lower intercept without visual fixation (p < 0.045). The first saccade showed a similar slope with and without visual fixation (p = 0.542), while the second and third saccades showed a steeper slope with visual fixation (p < 0.003). Comparing the saccade number, with visual fixation the first saccade showed a higher intercept at 1.0 compared to the second and third saccade (p < 0.008), while without visual fixation the first saccade was greater than the third saccade only (p = 0.001). All saccades showed a similar slope within each visual condition (p > 0.0213), except with visual fixation the slope of the second saccade was steeper than the first saccade (p = 0.0273).

#### Latency

Saccade onset latency showed a strongly correlated positive relationship (conditional r^2^ = 0.79, marginal r^2^ = 0.24) with the VOR gain (Fig 8); saccade latency decreased as VOR gain reduced, with a significant interaction of visual condition and saccade number (p = 0.003). Comparing visual conditions, the first saccade showed a lower intercept at 1.0 with visual fixation (p < 0.0001) but similar slope between visual conditions (p = 0.933), while the second showed a lower intercept (p = 0.0078) but shallower slope (p < 0.0001) without visual fixation (Fig 8). Comparing the saccade number, with visual fixation the order of intercepts at 1.0 order was first, second, then the third saccade (p < 0.0134). The first and second showed similar slopes (p = 0.231), while the third was shallower (p < 0.0011). Without visual fixation, the intercepts of all saccades at 1.0 were similar (p > 0.103), while the first saccade was steeper than the second and third saccade (p < 0.0007).

#### Clustering

Saccade latency SD also showed a positive relationship (conditional r^2^ = 0.77, marginal r^2^ = 0.12) with the VOR gain (Fig 8); thus, saccade clustering increased as the VOR gain reduced, with a significant interaction of visual condition and saccade number (p = 0.0093). Comparing visual conditions, all saccades showed a similar intercept at 1.0 (p > 0.372), but while the first saccade showed a steeper positive slope with visual fixation (p < 0.0003) the second saccade showed a slightly negative slope without visual fixation (p < 0.0378). The third saccade was similar between visual conditions (p > 0.372). Comparing the saccade number, with visual fixation the intercept at 1.0 of the first saccade was greater than second saccade (p = 0.0053) but showed a much steeper slope than the second and third saccade (p < 0.0001). The second and third saccade showed similar intercepts and slopes (p > 0.638). Without visual fixation, the second saccade showed a slightly lower intercept at 1.0 than the first saccade (p = 0.0373), but all saccades showed a similar slope (p > 0.193).

### 1.11 Saccade visual position error

#### The VOR gain

The visual position error of saccades in the light showed a strongly correlated negative relationship with the VOR gain (conditional r^2^ = 0.8471, marginal r^2^ = -0.431): as the VOR gain reduced the visual error increased. The first saccade showed significantly faster rate of increase in visual error than compared to later saccades (slope = 6.79(upper–lower CI: 10.94–2.64); p < 0.0459), while the second and third saccades were similar (slope = 2.77(6.92– -1.38); slope = 0.92(5.08– -3.25); p = 0.359).

#### Saccade amplitude

The visual position error of saccades in the light showed a very strongly correlated positive relationship with the amplitude of the subsequent saccade (conditional r^2^ = 0.975, marginal r^2^ = 0.047): as visual position error increased, the saccade amplitude also increased. Each saccade in the sequence (first: slope = 5.17(upper–lower CI: 17.90– -7.55), second: slope = 3.50(16.22– -9.66), third: slope = 2.75(15.50– -10.00)) showed similar rates of increase in amplitudes for the same visual error (p > 0.7031).

## Discussion

In this study, we investigated the effect of vision on the VOR gain and compensatory saccades in subjects with and without vestibular function. Bilaterally lesioned ears demonstrated the greatest changes in response to darkness, with a reduction in saccade amplitude, frequency, and clustering, and an increased latency. These results demonstrate that vision universally contributes to refixation saccades, however, the extent of this contribution is strongly dependent on residual vestibular function.

The presence of the VOR and saccades to maintain a gaze target (real or imaginary) can be thought of as compensatory velocity and position changes respectively [7, 22]. After the unilateral loss of the compensatory *velocity* response in UVD_ipsi_, we found subjects showed a new compensatory *position* response that depended on the *velocity* signal from the intact UVD_contra_ ear. While a single intact ear (UVD_ipsi_) was insufficient to generate a *velocity* change it was sufficient to generate a *position* change (Figs 1, 2 and 7), perhaps coordinated by separate velocity and position estimates [53]. After UVD the frequency and amplitude of the first saccade–often triggered during the head motion (‘covert saccades’) i.e. with dynamic vestibular input–showed little changes without vision (Figs 1 and 2). Thus, we could consider them an even more specific sign of ipsilateral vestibular loss than the more visually sensitive second and third saccades–usually triggered after the head comes to a stop (‘overt saccades’) i.e. without dynamic vestibular input [5, 25]. In summary, the first saccade could be thought as representing the vestibular *velocity* error while later saccades as representing the earlier saccade’s visual *position* error/s.

In contrast, after the bilateral loss of the compensatory velocity reflex in BVL, we found that subjects relied heavily in visual input–possibly either a position or velocity signal [54]–to generate the required position change, especially after the head stopped moving (Fig 1). Previous studies of compensatory saccades and posture after BVL also found an increased dependence on vision for position corrections [7, 22, 23, 55]. Surprisingly, we found that in the horizontal plane a single bilaterally deafferented (BVD) subject could still regularly generate early saccades without visual fixation (Fig 1), supporting a proprioceptive trigger [26], although these saccades were much smaller in amplitude and scattered in latency. Saccades were more affected in the vertical planes (Fig 1). Like BVD, saccades after BVL show a much greater dependence on visual fixation (Fig 3) [23]. The early latency, large amplitude, very frequent saccades generated by complete BVD *with* vision and complete UVD (UVD_ipsi_) *without* vision demonstrates that: for early saccades, residual vestibular input is just as adequate as visual input, and that early saccade triggering has sensory flexibility. The origin of early compensatory saccades therefore appears to be multi-sensory, dependent at least on any vestibular and visual inputs, a flexibility that highlights a key advantage over the VOR [26, 56].

As reported by numerous previous investigators [5, 7, 22, 23, 25, 57–61], we found that as the VOR loss increased compensatory saccade characteristics generally followed a similar pattern: the amplitude, frequency, and clustering increased while the latency became earlier (Fig 8). The rate of these changes to the reduction in VOR gain depended on both the visual condition and saccade number. Almost all characteristics of the first saccade strongly depended on vestibular function alone, as amplitudes and frequency increased, and latency decreased as the VOR gain reduced (Fig 8). However, in dark clustering remained unchanged by VOR gain loss, thus was related only to the visual condition. The presence of visual fixation enhanced saccade amplitude, frequency, and clustering, and decreased onset latency. The second saccade also showed dependence on both vestibular and visual function, however, this effect was weaker. Visual fixation increased saccade frequency, while amplitude, latency, and clustering showed a smaller effect. The third saccade showed the weakest dependence on both vestibular function and visual condition, with only frequency being affected by vision. Thus, the sequence of saccades differentially reflected an underlying multisensory dependence on vestibular function and visual conditions.

Comparing the effect of vision on the saccade number, we found that towards normal ears (normal and unlesioned UVD) the effect on the second saccade was generally similar or less compared to the first saccade. However, towards lesioned ears (lesioned UVD and BVL) the effect of vision on the second saccade was generally similar or more pronounced compared to the first saccade. Smaller effects reflect the already smaller amplitude, less frequent, and decreased clustering of the second saccade. This pattern probably reflects the underlying function of the second saccade to correct for the earlier first saccade, thus, after vestibular loss depends more on vision for the positional error signal.

Some studies have reported that the trial by trial variability in saccade latency (clustering) is related to vestibular compensation and rehabilitation [58, 59, 62]. We found that saccade clustering is strongly related to visual input but only weakly related to vestibular input (Fig 8), signifying that saccade clustering better represents visual substitution than vestibular compensation.

Without visual fixation normal subjects showed saccades that were small in amplitude, less frequent, and at longer latency compared to lesioned subjects. These saccade characteristics must change after vestibular loss to accomplish the goal to keep a stable gaze fixation, thereby proxy reflect the loss of VOR [4]. However, the negative effects of vision were significantly less in the earliest saccades to UVD_ipsi_ compared to BVL, such that these most likely represent a form of saccadic motor learning to a vestibular stimulus [53]. Sensori-motor learning of both the VOR and saccades requires time and exposure to errors [63, 64], suggesting that saccades characteristics could be of value in determining the age of a lesion e.g. useful when seeking to separate old and new vestibular lesions in sequential vestibular insults [65], as well as tracking vestibular rehabilitation intervention [57, 66, 67] and generally probing cognitive factors [68, 69] involved in compensating for the loss of a congenital reflex arc.

Previous studies have shown that saccades can rapidly become earlier in latency and larger in amplitude after unilateral and bilateral vestibular lesions [5, 25, 57, 58, 60, 70]. The rapid temporal clustering and reduction of overall saccade latency over time after vestibular loss indicates a simplification or reinforcement of the new stimulus-response relationship i.e. becoming more ‘reflexive’ [71, 72]. Even to passive impulsive head movements, subtle changes in visual fixation can modulate the VOR gain by a small degree [54, 71, 73], over multiple timescales [74], supplemented by the appearance of saccades. With different time scales of sensorimotor learning and forgetting in the VOR and saccadic systems, each provides different quantitative insight into the individual’s synthesis of the loss and their learning history [6].

This data builds on numerous previous reports [6, 22] and hypotheses [25, 56] that saccades have the capability to become a ‘conditioned-reflex’ or a ‘pseudo-reflex’ i.e. a response paired to a novel stimulus. A newly conditioned reflex, being conditional in nature [71], represents a more complex, temporary, learnt, and thus flexible relationship between stimulus and response, in contrast to the simpler, permanent, congenital, and thus less flexible reflex pathways [1, 75]. The VOR is truly reflexive, but while the saccadic system is not reflexive–indeed, can be modelled as a computational decision process [13, 14, 76]–still shows a close physiological relationship with the vestibular system [10, 20, 77] e.g. nystagmus during head rotations [78].

After VOR loss short latency compensatory saccades can improve visual acuity [79–82], so the VOR and saccades provide complimentary and differential roles in gaze by generating compensatory velocity and position changes respectively [7, 53]. The neuro-physiology of saccade modification after loss of the VOR could be explained by saccade motor learning paradigms, in man [83, 84] and monkey [85, 86], by which saccade characteristics are modulated over time by visual error feedback loops through the super colliculus and cerebellum [87–92].

In summary, the present study demonstrates that even healthy controls and the intact ears in UVD generate small refixation saccades. In lesioned ears, after the loss of the VOR, compensatory saccades provide a complimentary mechanism to reduce gaze error. Saccade frequency in healthy and lesioned ears is influenced by vision, decreasing when vision is denied. In lesioned ears, the effect of vision on saccade amplitude depends on the symmetry of the loss. After unilateral vestibular loss the remaining healthy ear can provide adequate input to trigger early compensatory saccades, however, bilateral vestibular loss results in saccades with a much greater dependence on vision. These observations could be extended when serially assessing the VOR after vestibular loss and may provide guidance and insight for quantification of vestibular rehabilitation.

## Supporting information

S1 Table Summary of subject data. The table follows tidy data principles, with each column a variable and each row an observation. Each numeric variable shows the mean±SD. SacNumber refers to the first and second saccade after the head impulse onset. SCC indicates the semicircular canal. NaN values indicate insufficient data to determine a value.
(CSV)
